# Vaccine herd effect

**DOI:** 10.3109/00365548.2011.582247

**Published:** 2011-05-23

**Authors:** Tae Hyong Kim, Jennie Johnstone, Mark Loeb

**Affiliations:** 1 Department of Pathology and Molecular Medicine, McMaster University, Hamilton, Ontario, Canada; 2 Division of Infectious Diseases, Departments of Internal Medicine, Soon Chun Hyang University, Seoul Hospital, Seoul, Republic of Korea; 3 Michael G. DeGroote Institute for Infectious Disease Research, McMaster University, Hamilton, Ontario, Canada; 4 Department of Medicine, McMaster University, Hamilton, Ontario, Canada

**Keywords:** Vaccine herd effect, vaccine herd immunity

## Abstract

Vaccination ideally protects susceptible populations at high risk for complications of the infection. However, vaccines for these subgroups do not always provide sufficient effectiveness. The herd effect or herd immunity is an attractive way to extend vaccine benefits beyond the directly targeted population. It refers to the indirect protection of unvaccinated persons, whereby an increase in the prevalence of immunity by the vaccine prevents circulation of infectious agents in susceptible populations. The herd effect has had a major impact in the eradication of smallpox, has reduced transmission of pertussis, and protects against influenza and pneumococcal disease. A high uptake of vaccines is generally needed for success. In this paper we aim to provide an update review on the herd effect, focusing on the clinical benefit, by reviewing data for specific vaccines.

## Introduction

**Figure 1:**
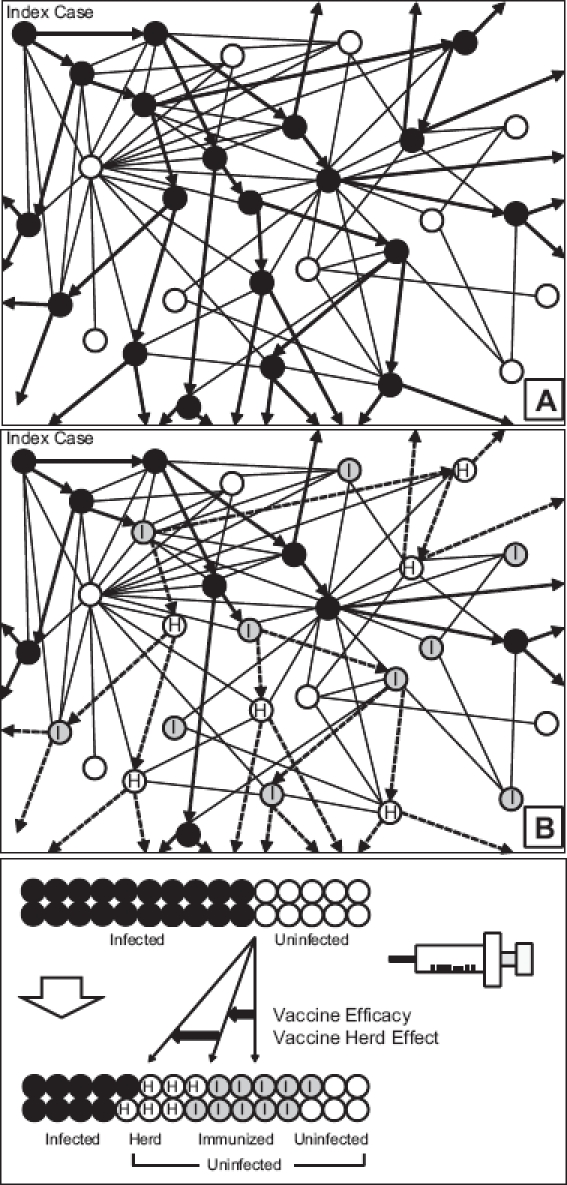
Schematic presentation of the herd effect: The index patient transmits an infectious agent to a given number (basic reproductive number *R*_0_) of susceptible persons in the community. Black circles are infected persons and white circles are uninfected susceptible persons (panel A). After a mass immunization programme in the community (panel B), a proportion of the population is immunized (grey circles with ‘I’), thus directly protected from the infectious agent (direct effect). The immunized individuals further protect the susceptible population (white circles with ‘H’) by stopping the transmission within the social networks. This extra protection effect provided by a vaccine is called ‘herd immunity’.

The direct effects of vaccination generally refer to the direct protection of the vaccinated individual, resulting in a reduced chance of infection and possibly complications. In contrast, the indirect benefits of vaccination refer to protective effects observed in unvaccinated populations [1]. This indirect effect of vaccination is known as the herd effect or ‘herd immunity’, defined as the indirect protection of unvaccinated persons, whereby an increase in the prevalence of vaccine-immunity prevents circulation of infectious agents in unvaccinated susceptible populations (Figure 1).

The importance of herd immunity was first recognized with smallpox, where the initial goal was to immunize 80% of the population in order to achieve such a herd effect. Although the ultimate eradication in 1977 was achieved with higher vaccine uptake rates, the herd effect contributed to the reduction of smallpox by a mass vaccination programme that focused on endemic countries [2]. Another important aspect of the herd effect is that it can play a key role in determining policy if it enhances cost-effectiveness. In the USA, it was estimated that the introduction of the quadrivalent meningococcal conjugate vaccine saved US$551 million in direct costs and $920 million in indirect costs, including costs associated with permanent disability and premature death [3]. Childhood pneumococcal vaccination is another example; the 7-valent pneumococcal conjugate vaccine was estimated to prevent 38,000 cases of invasive pneumococcal infection in the USA during its first 5 y of use at a cost of US$112,000 per life-y saved. However, the vaccine prevented 109,000 cases of invasive pneumococcal infection at a cost of $7500 per life-y saved when the herd effect was considered [4]. In the sections below, we review the experience of vaccination programmes and clinical trials in establishing a herd effect.

## Herd effects in Haemophilus influenzae type b vaccination

**Figure 2:**
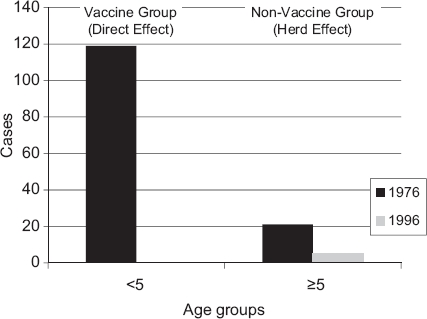
The vaccine herd effect on Haemophilus influenzae type b diseases in Finland after introduction of the Haemophilus influenzae type b conjugate vaccine in 1986 (adapted from Peltola et al. [5]).

Vaccination against invasive Haemophilus influenza type b with the conjugate vaccine began in high-income Scandinavian countries and resulted in a decline in invasive H. influenzae type b diseases in the vaccinated populations (0-4-y-olds: 49/100,000/y in 1986 to 0/100,000/y in 1996) with -95% effectiveness [5]. With a vaccine uptake of 50%, a herd effect appears to have occurred, as the reduction in invasive H. influenzae type b disease was also observed in unvaccinated older children (≥5 y) in Finland after the introduction of the H. influenzae type b conjugate vaccine in 1986 (Figure 2).

## Herd effects in pertussis vaccination

After the availability of pertussis vaccine in the 1940s, the introduction of pertussis immunization programmes with pertussis toxoid resulted in a sharp reduction in pertussis, not only among vaccinated infants, but also among non-vaccinated infants and older populations. For example, in a prospective study performed in Sweden, where the vaccine was only available after 1995 following an interruption of 16 y, there was a reduction in Bordetella pertussis isolation among non-vaccinated infants (1214 isolates between 1993 and 1995 to 64 isolates between 1997 and 1999; *p* < 0.0001). Moreover, pertussis-related hospitalization was reduced from 55 cases to 8 (population size 778,597) during the same time-periods (*p* < 0.0001) in the setting of an infant pertussis toxoid vaccine uptake of 56% [6]. It is of interest that herd protection after mass immunization eventually can wane over time, particularly where the immunogenicity of the vaccine does not allow for sustained antibody protection over time.This was evidenced by an outbreak of pertussis in Canada in 1990–1998 caused by a poorly protective whole cell vaccine [7].

It is sobering to note that after 30 y of intensive childhood immunization, there has been a recent re-emergence of adult pertussis, resulting in an increased risk of mortality in younger children [8]. The re-emergence of adult pertussis is likely due to a variety of factors, including waning vaccine immunity without a natural booster effect from household exposure, the loss of vaccine efficacy due to novel strains, and potentially also due to increased detection due to more sensitive diagnostic tests [9]. Reduced immunity among mothers has been particularly worrisome, as it has led to less effective transplacental immunity, increasing the vulnerability of young infants [10].

The majority of pertussis-related deaths occur in infants aged < 3 months, thus a series of vaccinations is now recommended for children (2,4,6,15-18 months, and 4-6 y in the USA [11] and Canada [12]). Using a polymerase chain reaction, culture, and serological examination, an observational study demonstrated that the likely source of infection in infants was siblings (41%), mothers (38%), and fathers (17%) [13]. Therefore, to better protect infants through the herd effect, a programme of booster pertussis vaccination with decennial tetanus toxoid, has been implemented in adults. The reduction of laboratory-confirmed pertussis in adolescents and adults by a booster adult vaccination was supported by a placebo-controlled randomized trial of acellular pertussis vaccine conducted in 2781 healthy adolescents and adults, showing a vaccine efficacy of 92% (95% confidence interval (CI) 0.32-0.99) [14]. Although evidence for the herd effect due to decennial administration of acellular pertussis vaccine in young adults leading to a reduction in B. pertussis mortality in children is not yet available, this programme is recommended in many countries [15].

## Herd effects in pneumococcal vaccination

Streptococcus pneumoniae causes both invasive (i.e. blood stream and other sterile sites) and non-invasive infection, such as community-acquired pneumonia. The burden of pneumococcal disease is high and is associated with significant morbidity and mortality. An estimated 1.6 million people, especially children aged < 5 y, die of invasive pneumococcal disease annually worldwide [16], thus prevention of this disease is of great importance. In addition, the optimal antibiotic therapeutic choices are restricted due to increasing resistance; therefore vaccines offer a potentially effective means to reduce invasive infections due to resistant strains. Currently there are 2 types of pneumococcal vaccine available: pneumococcal polysaccharide vaccines for adults and pneumococcal conjugate vaccines for children.

The efficacy of the pneumococcal polysaccharide vaccine was first identified in young African gold miners in 1977 [17]. Based on these data, and data from similar studies, the World Health Organization has recommended pneumococcal polysaccharide vaccine for people aged ≥ 65 y and those at increased risk of pneumococcal disease since the early 1980s. However, more recently there has been considerable debate about the efficacy and effectiveness of the 23-valent pneumococcal polysaccharide vaccine in this elderly population [18]. Although the pneumococcal polysaccharide vaccine prevents invasive pneumococcal disease (odds ratio (OR) 0.26, 95% CI 0.15–0.46), there is no evidence that it prevents all-cause pneumonia [19]. Moreover, another recent systematic review has questioned the efficacy of the pneumococcal polysaccharide vaccine in preventing pneumococcal pneumonia among the currently indicated populations (risk ratio (RR) 1.04, 95% CI 0.78–1.38) [20].

**Figure 3:**
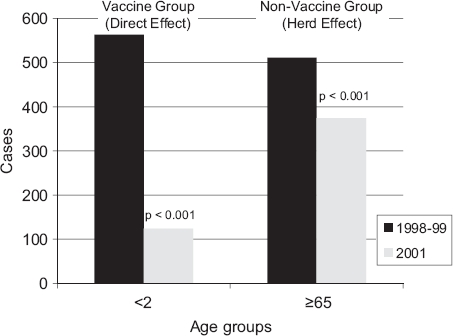
The vaccine herd effect on invasive pneumococcal diseases in the USA after introduction of the 7-valent pneumococcal conjugate vaccine in children aged < 2 y in 2000 (adapted from Whitney et al. [21]).

Evidence exists that the elderly have indirectly benefited from the introduction of the pneumococcal conjugate vaccine in children. The US Centers for Disease Control and Prevention Active Bacterial Core Surveillance (1996 to 2001) demonstrated a reduction in invasive pneumococcal disease in those aged ≥65 y after the introduction of the 7-valent pneumococcal conjugate vaccine, despite the fact that this population did receive the vaccine (Figure 3) [21]. Therefore, increasing the coverage of childhood pneumococcal conjugate vaccine will potentially further protect the elderly. As a result of childhood immunization with 7-valent pneumococcal conjugate vaccine, the Active Bacterial Core Surveillance data demonstrated a herd effect in the elderly and a reduction by 49% (16.4 to 8.4 cases per 100,000) of antimicrobial-resistant (penicillin non-susceptible S. pneumoniae) invasive pneumococcal disease [22]. It has been recognized that in these studies, the most resistant strains were 6B, 9V, 9 A, 14, 19F and 23F, all of which were covered by the 7-valent pneumococcal conjugate vaccine. However, the emergence of non-7-valent pneumococcal conjugate vaccine serotypes such as 19A [23], reveals the potential for serotype switching and in fact reverse the herd effect. Therefore, it is important that future vaccines increase the number of strains they target to reduce this possibility. Indeed, studies are now underway to determine the potential impact of the 13-valent pneumococcal conjugate vaccine on the herd effect, as the 13-valent vaccine has recently replaced the 7-valent vaccine in many countries.

## Herd effects in influenza vaccination

Seasonal influenza is a major cause of mortality resulting in an estimated 36,000 deaths annually in the USA alone [24]. The annual vaccination policy against seasonal influenza in most countries has been focused on protecting groups at high risk for complications of influenza, including the elderly, pregnant women, young children, and individuals with chronic diseases.

However, vaccinating high-risk populations is unlikely to reduce the burden of seasonal epidemics, because these groups represent only a fraction of the population among whom the virus spreads [25]. In addition, the attack rates in these groups are relatively low (8.8–13.5 per 100 persons for age ≥ 65 y). However, the attack rate is 25 per 100 persons in children aged 5–9 y and can reach 40 per 100 persons during pandemics, as experienced during 1918–19 [25].

Another challenge with only vaccinating high-risk groups is that the vaccines may not work as well in these at-risk populations. The efficacy of the influenza vaccine is dependent on the immunological status of the specific population being vaccinated and on the type of vaccine. For example, in healthy children, pooled estimates suggest that the live attenuated vaccine leads to a 79% efficacy (absolute risk reduction (ARR) 152.4 per 1000, number needed to treat (NNT) 6.6 persons) for reducing laboratory-confirmed influenza with 38% efficacy for reducing symptoms [26]. In contrast, in the elderly, no significant direct benefit of routine inactivated trivalent influenza vaccine was observed against influenza (RR 1.04, 95% CI 0.43–2.51) [26]. Of note, however, well-matched vaccines prevented complications in residents of long-term care facilities (vaccine efficacy (VE) of hospital admission 45%, 95% CI 0.16-0.64; all-cause mortality 60%, 95% CI 0.23–0.79) [27]. It is likely that the relative ineffectiveness of inactivated influenza vaccine in the elderly population is due to immune senescence, a waning of the immune system with age [28, 29].

**Figure 4:**
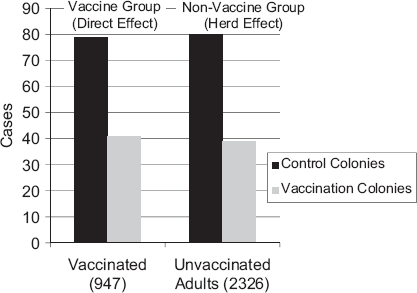
The vaccine herd effect on influenza in a randomized control trial in Canada (adapted from Loeb et al. [32]).

The fact that groups at the highest risk of complications from infection often benefit the least from the vaccine is an important public health and scientific challenge. Providing an indirect benefit to these groups by vaccinating those who respond well to vaccines is one way to mitigate this public health challenge. Data exist that a herd effect due to vaccination in children may help protect high-risk groups [30-32]. Recently, a cluster randomized study of trivalent inactivated influenza vaccination administered to 947 children and adolescents in Hutterites colonies in Canada showed a dramatic herd effect.The protective effectiveness in non-recipients of study vaccine was 61% (95% CI 0.08–0.83; *p* = 0.03) for reducing laboratory-confirmed influenza (3.1% in unvacci-nated adults of vaccinated colonies vs 7.6% in unvaccinated colonies; ARR 40.0 per 1000, NNT 25.0 persons; Figure 4) [32]. Such data lead to other important questions that still need to be answered, including the cost-effectiveness of vaccinating healthy children, the minimum uptake of vaccine in children needed to show a herd effect, and whether universal influenza vaccination is cost-effective. As has been demonstrated by Finnish researchers, the influenza vaccine is cost-effective when administered to children aged 6–13 y when considering the direct benefits of the vaccine [33]. Since there is now evidence that a benefit of up to 60% effectiveness may be seen in unvaccinated individuals due to the herd effect [32], this argues for an even greater cost-effectiveness of immunizing children.

The Society for Healthcare Epidemiology of America recently released a statement that influenza vaccination of healthcare personnel is a core patient and healthcare personnel safety practice [34]. Whether vaccination of healthcare personnel can lead to a herd effect reducing laboratory-confirmed influenza among patients is still inconclusive. Pooled data from a Cochrane review of 3 cluster randomized controlled trials showed no reduction of laboratory-confirmed influenza (OR 0.86, 95% CI 0.44–1.68; *p* = 0.66), lower respiratory tract infections, admission to hospital (OR 0.89, 95% CI 0.75–1.06), and deaths from pneumonia (OR 0.82, 95% CI 0.45–1.49) in patients when healthcare personnel were vaccinated [35]. However, given that mathematical models suggest a herd effect [36], more rigorous studies need to be conducted.

## Herd effects in meningococcal vaccination

Meningococcal meningitis causes devastating epidemics in sub-Saharan Africa where vaccine prevention is most needed. Because most meningococcal infections are caused by 6 of the 13 known serogroups (A, B, C, W-135, X, Y), these epidemics are preventable by vaccine. In 1991, a school-based cluster randomized double-blind trial of a serogroup B meningococcal vaccine that involved 171,800 students resulted in a vaccine efficacy of 71% [37]. Although this trial was performed in Norway, which had a high incidence of serogroup B meningococcal disease [38, 39], it failed to demonstrate a reduction in meningococcal disease among unvaccinated children in the vaccinated clusters. Evidence of a vaccine herd effect was suggested after adoption of routine monovalent serogroup C meningococcal vaccination in infants in England in 1999, where the incidence of meningococcal serogroup C disease declined not only in the vaccine group, but also in unvaccinated groups (by 67% in those aged 1–17 y and 35% in those aged >25 y) [40]. During the same period, carriage of serogroup C meningococci was reduced by 66% (*p* = 0.004) according to data collected from 14,064 students aged 15–17 y at the time of vaccination, and 16,583 students 1 y later [41].

Although nasal carriage is the basic step for invasive infection, the relationship between acquisition (carriage) and infection is not yet clear. Neisseria meningitidis commonly (∼10%) colonizes the human oropharyngeal mucosa, and asymptomatic carriage is perennial occurring with a high frequency in teenagers where there is substantial genetic diversity of strains that are isolated [42]. Symptomatic infections on the other hand are seasonal and occur more commonly in younger children [42]. Quadrivalent (A, C, Y, W-135) vaccines are licensed in the USA for those aged 11–18 y and in persons aged 2–55 y who are at elevated risk for invasive meningococcal disease [43]. Active surveillance in the USA for invasive N. meningitidis during 1998–2007 showed a 64.1% reduction in the annual incidence, from 0.92 cases per 100,000 population in 1998 to 0.33 cases per 100,000 population in 2007 [44].

## Herd effects in rotavirus vaccination

Rotavirus is now the most important cause of gastroenteritis in young children (age < 5 y) [45]. Since natural infection caused by a single serotype in infancy results in protection against subsequent infection by both homotypic and heterotypic viruses [46], vaccination against certain serotypes alone might reduce the burden of rotavirus infection. Several vaccines have proven efficacy and safety [47].

In addition, a herd benefit of rotavirus vaccine is suggested by laboratory-based surveillance data [48] and mathematical modelling [49]. With an estimated vaccination rate of ∼50% in the USA with pentavalent rotavirus vaccine, an 87% reduction in cases was observed from the hospital-based population surveillance during the outbreak seasons following introduction of the vaccine. According to the mathematical models, the predicted additional protection of rotavirus-related gastroenteritis by vaccine herd effect was 25%, 22%, and 20% with vaccine uptake rates of 70%, 90%, and 95%, respectively. A herd benefit was also suggested in Nicaragua by observing the reduction of acute gastroenteritis following adoption of free rotavirus vaccine for all eligible children [50]. The World Health Organization recently recommended that rotavirus vaccine be included in the immunization programmes of countries where data on vaccine efficacy suggest a significant public health impact. However, since the highest mortality rates occur in sub-Saharan Africa and South Asia, further evidence of direct and indirect effects should be sought in these countries [51].

## Potential herd effects in human papillomavirus (HPV) vaccination

Human papillomavirus (HPV), although not a notifiable disease, is considered the most common agent of sexually transmitted infection given the high global incidence of HPV DNA in sexually active women [52]. A meta-analysis of 157,879 women with normal cervical cytology approximates the prevalence of HPV DNA to be 10.4% (95% CI 10.2–10.7%) [53]. Persistent HPV infection is the greatest risk factor for the development of high-grade precancerous lesions or invasive cervical cancer. The burden of HPV infection, along with cervical cancer mortality, appears to be even higher in developing countries [54]. Even within the context of a decreasing incidence of cervical cancer in resource-rich countries, where effective screening programmes and promotion of condom use are in place, vaccination against certain subtypes of HPV has proven effective in the prevention of the HPV infection and precancerous cervical disease [55]. The direct effect was supported by a meta-analysis of 6 randomized controlled studies, showing a reduced frequency of high-grade cervical lesion by an OR of 0.14 (95% CI 0.09–0.21) [56].

Although these vaccines are recommended in developed countries, the field efficacy and indirect benefits, including the herd effect of HPV vaccination, remain unknown. There is still debate about the cost-effectiveness of such expensive vaccines compared to the usual screening and promotion of condom use in resource-limited countries [57]. However, with the high efficacy of the vaccine against cervical cancer, a vaccine herd effect might be expected, especially in high endemic regions. At present, a benefit has only been shown through mathematical modelling [58]. Moreover, the effect of a vaccine herd effect in women by vaccinating men, or vice versa, has not yet been proven.

## Conclusions

In summary, we have shown that the benefits of many current vaccines extend beyond the direct benefits to indirect benefits, i.e. through the herd effect extending beyond targeted groups to other populations at high risk for complications. Nevertheless, gaps in our knowledge exist about how best to achieve herd immunity. For example, it is unclear whether there are particular formulations that confer better herd immunity than others; a prime example is whether herd immunity achieved through live attenuated influenza vaccine is superior to that achieved with inactivated vaccine. Another area where gaps in our knowledge exist is the optimal use of new vaccines. For example, there are several candidate vaccines for dengue in clinical trials and strategies for how best to use them to establish herd immunity need to be developed.
